# Reduced decline of lung diffusing capacity in COPD patients with diabetes and metformin treatment

**DOI:** 10.1038/s41598-022-05276-x

**Published:** 2022-01-26

**Authors:** Kathrin Kahnert, Stefan Andreas, Christina Kellerer, Johanna I. Lutter, Tanja Lucke, Önder Yildirim, Mareike Lehmann, Jochen Seissler, Jürgen Behr, Marion Frankenberger, Robert Bals, Henrik Watz, Tobias Welte, Franziska C. Trudzinski, Claus F. Vogelmeier, Peter Alter, Rudolf A. Jörres, COSYCONET Study Group, Stefan Andreas, Robert Bals, Jürgen Behr, Kathrin Kahnert, Thomas Bahmer, Burkhard Bewig, Ralf Ewert, Beate Stubbe, Joachim H. Ficker, Christian Grohé, Matthias Held, Markus Henke, Felix Herth, Anne-Marie Kirsten, Henrik Watz, Rembert Koczulla, Juliane Kronsbein, Cornelia Kropf-Sanchen, Christian Herzmann, Michael Pfeifer, Winfried J. Randerath, Werner Seeger, Michael Studnicka, Christian Taube, Hartmut Timmermann, Peter Alter, Bernd Schmeck, Claus Vogelmeier, Tobias Welte, Hubert Wirtz

**Affiliations:** 1 Department of Medicine V, Comprehensive Pneumology Center, Member of the German Center for Lung Research (DZL), University Hospital, LMU Munich, Ziemssenstraße 1, 80336 Munich, Germany; 2 LungClinic Immenhausen, Member of the German Center for Lung Research (DZL), Immenhausen, Germany; 3 Institute and Outpatient Clinic for Occupational, Social and Environmental Medicine, Comprehensive Pneumology Center Munich (CPC-M), Member of the German Center for Lung Research (DZL), University Hospital of Munich (LMU), Munich, Germany; 4 School of Medicine, Institute of General Practice and Health Services Research, Technical University of Munich (TUM), Munich, Germany; 5 Institute of Health Economics and Health Care Management, Helmholtz Zentrum München GmbH - German Research Center for Environmental Health, Comprehensive Pneumology Center Munich (CPC-M), Member of the German Center for Lung Research, Munich, Germany; 6 Institute of Lung Biology and Disease (ILBD), Comprehensive Pneumology Center Munich (CPC-M), Member of the German Center for Lung Research (DZL), Munich, Germany; 7 Department of Medicine IV, Diabetes Center, University Hospital, LMU Munich, Munich, Germany; 8 Department of Internal Medicine V – Pulmonology, Allergology, Respiratory Intensive Care Medicine, Saarland University Hospital, Homburg, Germany; 9 Pulmonary Research Institute at LungenClinic Grosshansdorf, Airway Research Center North (ARCN), Member of the German Center for Lung Research (DZL), Grosshansdorf, Germany; 10 Department of Pneumology, Hannover Medical School, Hannover, Germany; 11 Thoraxklinik Heidelberg, Translational Lung Research Center Heidelberg (TLRC-H), Member of the German Center for Lung Research (DZL), University Hospital of Heidelberg, Heidelberg, Germany; 12 Department of Medicine, Pulmonary and Critical Care Medicine, Member of the German Center for Lung Research (DZL), University Medical Center Giessen and Marburg, Philipps-University Marburg (UMR), Marburg, Germany; 13 Universitätsklinikum Schleswig Holstein, Kiel, Germany; 14 Städtisches Krankenhaus Kiel, Kiel, Germany; 15 Universitätsmedizin Greifswald, Greifswald, Germany; 16 Klinikum Nürnberg, Paracelsus Medizinische Privatuniversität Nürnberg, Nuremberg, Germany; 17 Ev. Lungenklinik Berlin, Berlin, Germany; 18 Klinikum Würzburg Mitte gGmbH, Standort Missioklinik, Würzburg, Germany; 19 Asklepios Fachkliniken München-Gauting, Gauting, Germany; 20 Thoraxklinik Heidelberg gGmbH, Heidelberg, Germany; 21 Schön Klinik Berchtesgadener Land, Schönau am Königssee, Germany; 22 Berufsgenossenschaftliches Universitätsklinikum Bergmannsheil, Bochum, Germany; 23 Universitätsklinikum Ulm, Ulm, Germany; 24 Forschungszentrum Borstel, Borstel, Germany; 25 Klinik Donaustauf, Donaustauf, Germany; 26 Wissenschaftliches Institut Bethanien e. V., Solingen, Solingen, Germany; 27 Justus-Liebig-Universität Gießen, Giessen, Germany; 28 Uniklinikum Salzburg, Salzburg, Austria; 29 Ruhrlandklinik gGmbH Essen, Essen, Germany; 30 Hamburger Institut für Therapieforschung GmbH, Hamburg, Germany; 31 Universitätsklinikum Leipzig, Leipzig, Germany

**Keywords:** Epidemiology, Drug therapy

## Abstract

We studied whether in patients with COPD the use of metformin for diabetes treatment was linked to a pattern of lung function decline consistent with the hypothesis of anti-aging effects of metformin. Patients of GOLD grades 1–4 of the COSYCONET cohort with follow-up data of up to 4.5 y were included. The annual decline in lung function (FEV_1_, FVC) and CO diffusing capacity (KCO, TLCO) in %predicted at baseline was evaluated for associations with age, sex, BMI, pack-years, smoking status, baseline lung function, exacerbation risk, respiratory symptoms, cardiac disease, as well as metformin-containing therapy compared to patients without diabetes and metformin. Among 2741 patients, 1541 (mean age 64.4 y, 601 female) fulfilled the inclusion criteria. In the group with metformin treatment vs. non-diabetes the mean annual decline in KCO and TLCO was significantly lower (0.2 vs 2.3, 0.8 vs. 2.8%predicted, respectively; *p* < 0.05 each), but not the decline of FEV_1_ and FVC. These results were confirmed using multiple regression and propensity score analyses. Our findings demonstrate an association between the annual decline of lung diffusing capacity and the intake of metformin in patients with COPD consistent with the hypothesis of anti-aging effects of metformin as reflected in a surrogate marker of emphysema.

## Introduction

There is increasing evidence that chronic obstructive pulmonary disease (COPD) is partially driven by accelerated lung aging, compared to cigarette smokers without airway obstruction or non-smokers^1–3^. Moreover, COPD patients frequently have comorbidities that are also associated with aging, for example atherosclerosis, type 2 diabetes mellitus, or chronic kidney failure^4,5^. Cellular senescence, as biological correlate of aging, can compromise the innate and adaptive immune defence. It also includes the accumulation of senescent cells in the lung comprising airway and alveolar epithelial cells, vascular endothelial cells, and fibroblasts^6–9^. Several molecular pathways, including the activation of phosphoinositide 3-kinase (PI3K) or Mammalian Target of Rapamycin (mTOR), contribute to cellular aging. Accordingly, markers of aging, such as the activity of cell cycle regulators or telomere length, have been found to be altered in the lung of patients with COPD^10^, suggesting that premature aging is one of the factors underlying COPD and emphysema^11^.

This spurred research into the molecular pathways of lung aging, including the identification of potential novel drug targets or beneficial side-effects of known compounds. A rationale for the present analysis was the previous finding in a cross-sectional analysis that COPD patients with diabetes mellitus had no worse carbon monoxide (CO) diffusing capacity than patients without diabetes^12^, although one should have expected lower values due to additional vascular damage from diabetes; surprisingly, there was even a tendency towards better values. This might indicate an association between COPD phenotype (emphysema vs. airway-dominated) and the risk for diabetes^13^, or potential protective effects of anti-diabetic medication against emphysema^12^. Among anti-diabetic medication, metformin is known since decades and widely used. Its range of effects includes activation of adenosine monophosphate-activated protein kinase (AMPK), an endogenous mTOR inhibitor, thereby reducing cellular senescence and its associated secretory profiles (SASP)^14,15^. Although several studies have been dedicated to the identification of effects of metformin on senescence-associated processes in both cell culture and animal models^16–19^, clinical data are rare. Two recent studies reported a reduction of mortality in COPD patients taking metformin^20,21^, moreover another large study showed an association between metformin use in type 2 diabetes and a significant decrease in the risk of mortality from chronic lower respiratory disease^22^. Furthermore, there are very recent data on lesser emphysema progression over time in patients with COPD taking metformin^19^. We hypothesized that the beneficial effect of metformin might also be manifest in a reduced decline of emphysema-related functional markers over time, especially CO diffusing capacity, in contrast to functional markers less closely linked to emphysema.

Based on these considerations, we studied whether the intake of metformin in patients with type 2 diabetes and COPD was associated with the time course of lung function. Data were obtained from COSYCONET (COPD and Systemic Consequences—Comorbidities Network), a large, multi-center cohort study of COPD patients, and the statistical tools comprised multiple regression analysis and propensity score matching.

## Methods

### Study population

Data from patients of GOLD grades 1–4^23^ obtained at visit 1 as well as follow-up data from visits 2, 3, 4 and 5 of the COSYCONET cohort were used^24^; these visits were scheduled 0.5, 1.5, 3 and 4.5 years after recruitment. For each visit, patients were selected who had valid data on FEV_1_, FVC, KCO, TLCO, pack-years and smoking status, GOLD groups (based on mMRC) and spirometric grades, BMI, cardiovascular comorbidities, diabetes with continuous metformin treatment over all study visits, or no diabetes. Specifically, visit 1 of COSYCONET comprised n = 2741 patients, of whom 450 did not meet the criterion FEV_1_/FVC < 0.7 and were excluded. Further 172 patients were excluded due to missing or invalid data on GOLD groups A-D (based on mMRC), BMI, packyears or smoking status. Another 432 patients with incomplete data for FEV_1_, FVC, KCO, or TLCO were also excluded, resulting in n = 1687 patients. To identify the association of metformin with COPD progression as clearly as possible, diabetes patients without metformin therapy (including dietary measures alone or other anti-diabetic medication), and patients without continuous metformin therapy across all follow-up visits were excluded (n = 146 excluded). This resulted in a final study population of n = 1541 patients (n = 76 diabetes patients with continuous metformin treatment and n = 1465 non-diabetes patients. See Fig. S1). Among these patients, n = 186, 392, 248 and 715 had their last measurements in visits 2, 3, 4 and 5, respectively. The COSYCONET study was approved by the Ethical Committees of all study centers, and all patients gave their written informed consent^24^ (trial registration NCT0124593). The COSYCONET study was conducted in accordance with the Declaration of Helsinki. All methods were performed in accordance with relevant guidelines.

### Assessments

Study protocol and assessments of COSYCONET have been described previously^24^. Comorbidities were identified from patients’ reports of physician-based diagnoses in combination with disease-specific medication^25^. Inhaled and oral medication was recorded at each visit following a standard procedure^25^. The presence of cardiac comorbidities was indicated by a combined variable including heart failure, coronary artery disease and of history of myocardial infarction. The assessment of COPD symptoms and exacerbations followed GOLD criteria^23^, with symptoms rated according to mMRC (modified Medical Research Council). We also used GOLD groups AC versus BD as binary symptoms score, and groups AB versus CD as binary exacerbation score. Glycated hemoglobin (HbA1c) was assessed following standardized operating procedures^24^. Spirometric data comprised forced expiratory volume in 1 s (FEV_1_) and forced vital capacity (FVC) in percent predicted, while carbon monoxide (CO) diffusing capacity included the single-breath diffusing capacity (TLCO) and the transfer coefficient (KCO). All measurements followed international and national recommendations as implemented in the study protocol^24^. Predicted values of spirometric measures were taken from the Global Lung Initiative (GLI)^26^, as well as those for diffusing capacity^27^.

### Statistical analysis

Data are presented as numbers and percentages, or mean values and standard deviations (SD). Comparisons between groups (patients with diabetes and metformin versus patients without diabetes) were performed by analysis of variance (two groups, equivalent to t-test), or by chi-square-statistics in case of categorical variables. The calculation of annual lung function decline was based on the difference between values obtained at the patient's last visit and baseline visit; this difference was divided by the number of years between the two visits and expressed in terms of %predicted at baseline.

Associations between variables were identified by linear regression analyses comprising multiple independent predictors and one dependent variable. Age, sex, BMI, pack-years and smoking status were always included as predictors, moreover exacerbation history and symptoms, the presence of cardiac disease, all of them at baseline (visit V1), as well as baseline FEV_1_ (%predicted). These variables were selected as potentially relevant predictors from a pathophysiological and clinical point of view. The dependent variables were the annual changes of FEV_1_, FVC, KCO and TLCO in separate analyses. For each of the annual changes, the respective baseline value was included as further predictor, except for FVC due to its high correlation with FEV_1_. The target predictor was the presence of diabetes therapy containing metformin across all study visits of the individual patients. In sensitivity analyses, HbA1c and hemoglobin were included as additional predictors, as well as the number of the final visit as categorical variable.

To check the results with an alternative approach, propensity score analysis was used as a procedure for matching the groups of patients without diabetes and diabetes with metformin, similarly to a recent work on associations of COPD therapy with left heart parameters^28^. Propensity scores were determined using logistic regression analysis followed by full and genetic matching; the predictors used were the same as in the conventional regression analyses. The two methods served as additional check of the robustness of the results, since there are several established, not necessarily equivalent methods of matching^29^. Full matching works by potentially assigning several patients of the reference group to the treatment group and expressing their relative importance by statistical weights, whereas genetic matching aims to achieve the same distribution of propensity scores by an iterative selection procedure. The effect estimates from matching were then derived by linear regression analysis including the propensity scores and all predictors mentioned above. The efficiency of matching was quantified by the standardized mean differences between groups for each variable, which should not be greater than 0.25, ideally 0.1.

All analyses were performed with the software IBM SPSS Statistics (Version 26, IBM Corp., Armonk, NY, USA). For propensity score analysis, R (Version 4.0.2) was used. Propensity scores were computed using the package “dplyr”, matching was performed via the packages “MatchIt” and “optmatch, and outcome evaluation by weighted regression via the package “survey”. P values less than 0.05 were considered as significant.

### Ethics approval and consent to participate

All assessments were approved by the central (Marburg (Ethikkommission FB Medizin Marburg) and local (Bad Reichenhall (Ethikkommission bayerische Landesärztekammer); Berlin (Ethikkommission Ärztekammer Berlin); Bochum (Ethikkommission Medizinische Fakultät der RUB); Borstel (Ethikkommission Universität Lübeck); Coswig (Ethikkommission TU Dresden); Donaustauf (Ethikkommission Universitätsklinikum Regensburg); Essen (Ethikkommission Medizinische Fakultät Duisburg-Essen); Gießen (Ethikkommission Fachbereich Medizin); Greifswald (Ethikkommission Universitätsmedizin Greifswald); Großhansdorf (Ethikkommission Ärztekammer Schleswig–Holstein); Hamburg (Ethikkommission Ärztekammer Hamburg); MHH Hannover/Coppenbrügge (MHH Ethikkommission); Heidelberg Thorax/Uniklinik (Ethikkommission Universität Heidelberg); Homburg (Ethikkommission Saarbrücken); Immenhausen (Ethikkommission Landesärztekammer Hessen); Kiel (Ethikkommission Christian-Albrechts-Universität zu Kiel); Leipzig (Ethikkommission Universität Leipzig); Löwenstein (Ethikkommission Landesärztekammer Baden-Württemberg); Mainz (Ethikkommission Landesärztekammer Rheinland-Pfalz); München LMU/Gauting (Ethikkommission Klinikum Universität München); Nürnberg (Ethikkommission Friedrich-Alexander-Universität Erlangen Nürnberg); Rostock (Ethikkommission Universität Rostock); Berchtesgadener Land (Ethikkommission Land Salzburg); Schmallenberg (Ethikkommission Ärztekammer Westfalen-Lippe); Solingen (Ethikkommission Universität Witten-Herdecke); Ulm (Ethikkommission Universität Ulm); Würzburg (Ethikkommission Universität Würzburg)) Ethical Committees, and written informed consent was obtained from all patients. The study was based on 2741 patients recruited within the COSYCONET framework (ClinicalTrials.gov, Identifier: NCT01245933). For further information see Karch et al.^24^.

### Consent for publication

Within the ethical approval, the participants of the study gave their consent to publish the data collected during the study period.

## Results

### Baseline characteristics

**Figure 1:**
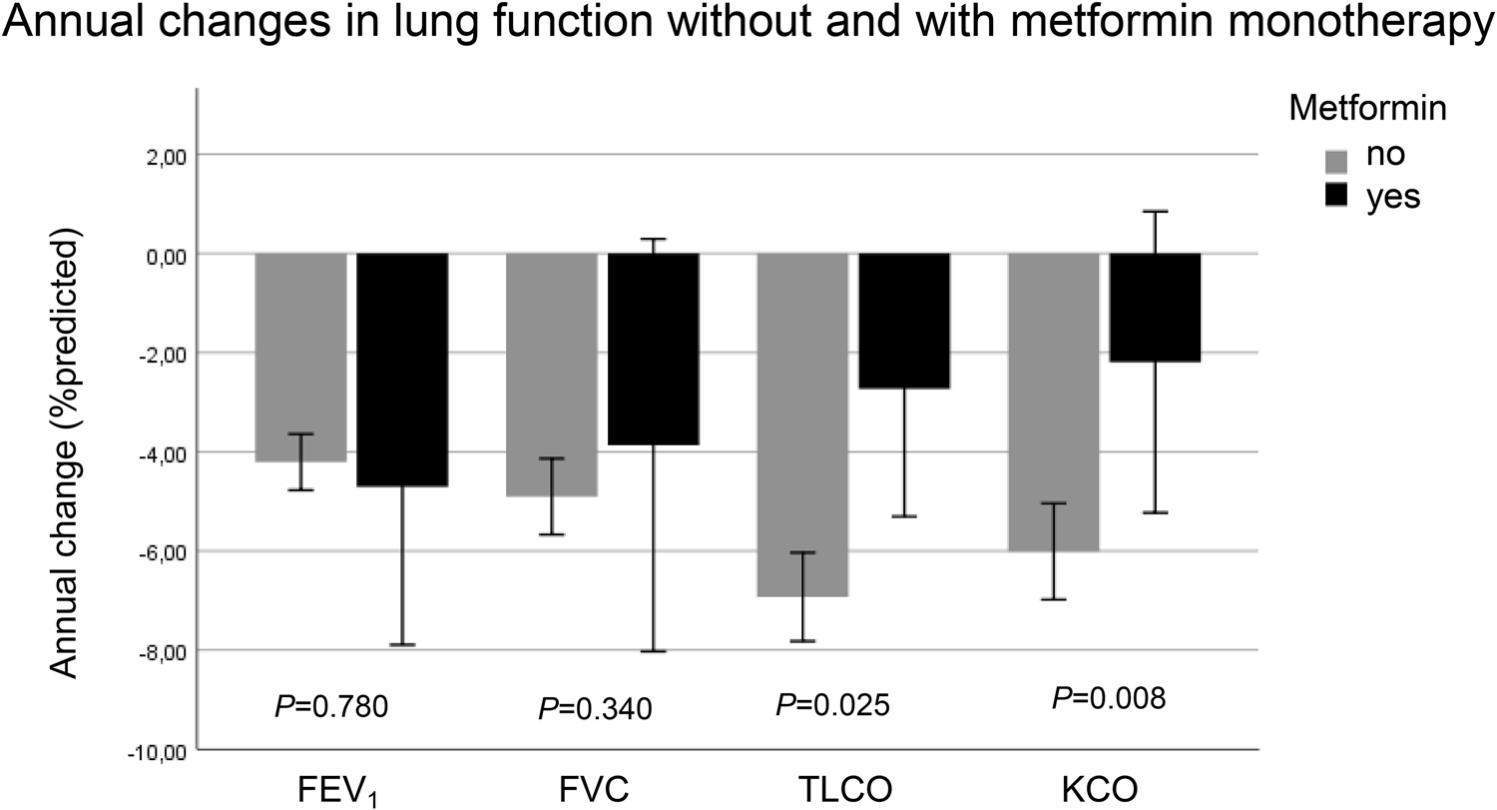
Annual changes in lung function measures. Unadjusted annual changes in lung function measures (mean and 95%-confidence intervals) for the two groups receiving either no metformin (light bars) or metformin as continuous therapy (dark bars). Units are %predicted relative to baseline (GLI). For adjusted values see Table 2.

Among 2741 patients included at visit 1, 385 (14%) had the diagnosis of diabetes, and 182 (47% of diabetes patients) took metformin at visit 1. The numbers of patients for whom the subsequent visits V2–V5 were the final visits, were n = 360, 603, 416 and 1046. When requiring complete data regarding GOLD grades 1–4, GOLD groups A–D, smoking status, pack-years, FEV_1_, FVC, TLCO and KCO, as well as including only patients with continuous metformin therapy over all visits, this resulted in a study population of n = 1541 patients, of whom n = 186, 392, 248 and 715 patients had data at their last visits V2–V5, respectively (Fig. S1, Table 1). Patients with diabetes and continuous metformin therapy (n = 76) and patients without diabetes (n = 1465) showed significant differences regarding sex, cardiac disease, BMI, HbA1c, pack-years, TLCO and KCO (*p* < 0.05 each, see Table 1). The annual decline of TLCO and KCO was also lower in patients with metformin treatment vs non-diabetes patients (*p* < 0.05 each), whereas there were no significant differences regarding FEV_1_ and FVC; these unadjusted values are shown in Fig. 1.

**Table 1:** Baseline characteristics.

Variable	All (n = 1541)	Control (n = 1465)	Metformin (n = 76)
Sex (m/f)	940/601 (60.1%/39.0%)	871/594 (59.5%/40.5%)	69/7*** (90.8%/9.2%)
Age (years)	64.4 ± 8.3	64.3 ± 8.3	65.9 ± 7.3
BMI (kg/m^2^)	26.6 ± 5.2	26.4 ± 5.0	30.0 ± 4.8***
Smoking status (never/ex vs. active)	1129/412	1078/387	51/25
Pack-years	49.0 ± 36.0	48.3 ± 35.0	62.8 ± 48.7***
FEV_1_ (%predicted)	54.2 ± 18.0	54.1 ± 18.1	57.0 ± 15.3
FVC (%predicted)	80.5 ± 18.4	80.6 ± 18.6	78.4 ± 16.1
KCO (%predicted)	63.5 ± 21.3	63.1 ± 21.2	71.2 ± 21.3***
TLCO (%predicted)	56.1 ± 21.0	55.8 ± 20.9	62.1 ± 20.6**
Hba1c (%)	5.81 ± 0.53	5.7 ± 0.4	6.9 ± 0.7***
CRP (mg/dl)	1.02 ± 3.10	1.0 ± 3.2	1.0 ± 1.7
Creatinine (mg/dl)	0.88 ± 0.21	0.88 ± 0.22	0.90 ± 0.18
GOLD grades 1/2/3/4	150/686/582/123	145/642/557/121	5/44/25/2
GOLD groups A/B/C/D	657/367/206/302	624/352/201/288	33/24/5/14
Diabetes with continuous metformin treatment (yes/no)	76/1465	–	–
Cardiovascular disease (yes/no)^a^	290/1251	265/1200	25/51***

The table shows the baseline characteristics of the study cohort. For continuous variables, mean values and standard deviations are given. Furthermore, categorical data for sex, smoking status, GOLD grades and groups, diagnosis of diabetes, diagnosis of cardiovascular disease and diabetes with continuous metformin treatment are given. For abbreviations see “Methods” section.

^a^The diagnosis of cardiovascular disease comprised heart failure, coronary artery disease and myocardial infarction. This combination was chosen as the case numbers of the single items were low.

**p < 0.01, ***p < 0.001.

### Relationship between functional parameters and metformin treatment

Among the covariates age, sex, BMI, smoking status, pack-years, FEV_1_, symptoms (GOLD groups BD according to mMRC), exacerbations (GOLD groups CD), cardiac disease and treatment with metformin-containing therapy, only the variables BMI, smoking status and baseline FEV_1_ showed a significant association with the annual decline of FEV_1_ (*p* < 0.05 each); there was no significant association with metformin. Repeating the analysis for the annual decline of FVC, there was only a tendency for baseline symptoms to be associated with the annual decline (*p* = 0.056). In contrast, for the annual decline of KCO, the variables BMI, pack-years, symptoms, baseline FEV_1_ and KCO were significant predictors (*p* < 0.05), in addition to metformin therapy (*p* = 0.009) (Table 2). Similarly, when the annual decline of TLCO was analyzed, metformin therapy was significantly (*p* = 0.005) related to the decline, in addition to age, BMI, pack-years, symptoms, baseline FEV_1_ and TLCO (*p* < 0.05).

**Table 2:** Association between annual decline of KCO %predicted and metformin monotherapy.

Predictors	Non-standardized		Standardized coefficient	*p* value	95%-Confidence interval for B		Collinearity VIF
	Regression coefficient B	SE	Beta		Lower	Upper	
Sex (female vs. male)	0.348	0.420	0.021	0.407	− 0.475	1.172	1.120
Age (y)	− 0.047	0.025	− 0.049	0.058	− 0.095	0.002	1.104
BMI (kg/m^2^)	0.223	0.043	0.142	< 0.001	0.139	0.308	1.260
Pack-years	− 0.023	0.006	− 0.102	< 0.001	− 0.034	− 0.011	1.162
Smoking status (active)	− 0.786	0.475	− 0.044	0.098	− 1.719	0.146	1.182
Symptoms (GOLD BD vs AC)	− 1.062	0.441	− 0.066	0.016	− 1.927	− 0.197	1.279
Exacerbations (GOLD CD vs AB)	0.246	0.430	0.015	0.567	− 0.598	1.091	1.093
Cardiovascular disease*	0.596	0.512	0.029	0.245	− 0.409	1.601	1.071
FEV_1_%predicted baseline	0.092	0.013	0.208	< 0.001	0.067	0.116	1.364
KCO %predicted baseline	− 0.103	0.011	− 0.276	< 0.001	− 0.124	− 0.082	1.358
Metformin therapy (continuous)	2.413	0.918	0.066	0.009	0.613	4.213	1.054

The table shows the results of multivariate linear regression analysis in terms of the non-standardized regression coefficients, their standard errors (SE), and 95%-confidence intervals, and the standardized coefficients. All clinical and functional indices refer to baseline (visit 1), the change of KCO to that between baseline and each patient’s last visit, expressed as %predicted at baseline. Additionally, the Variance Inflation Factor (VIF) from the collinearity diagnostics in SPSS is given, indicating that there was no problem with collinearity as all values were close to 1.

*The diagnosis of cardiovascular disease comprised heart failure, coronary artery disease and myocardial infarction.

### Propensity score analysis

**Figure 2:**
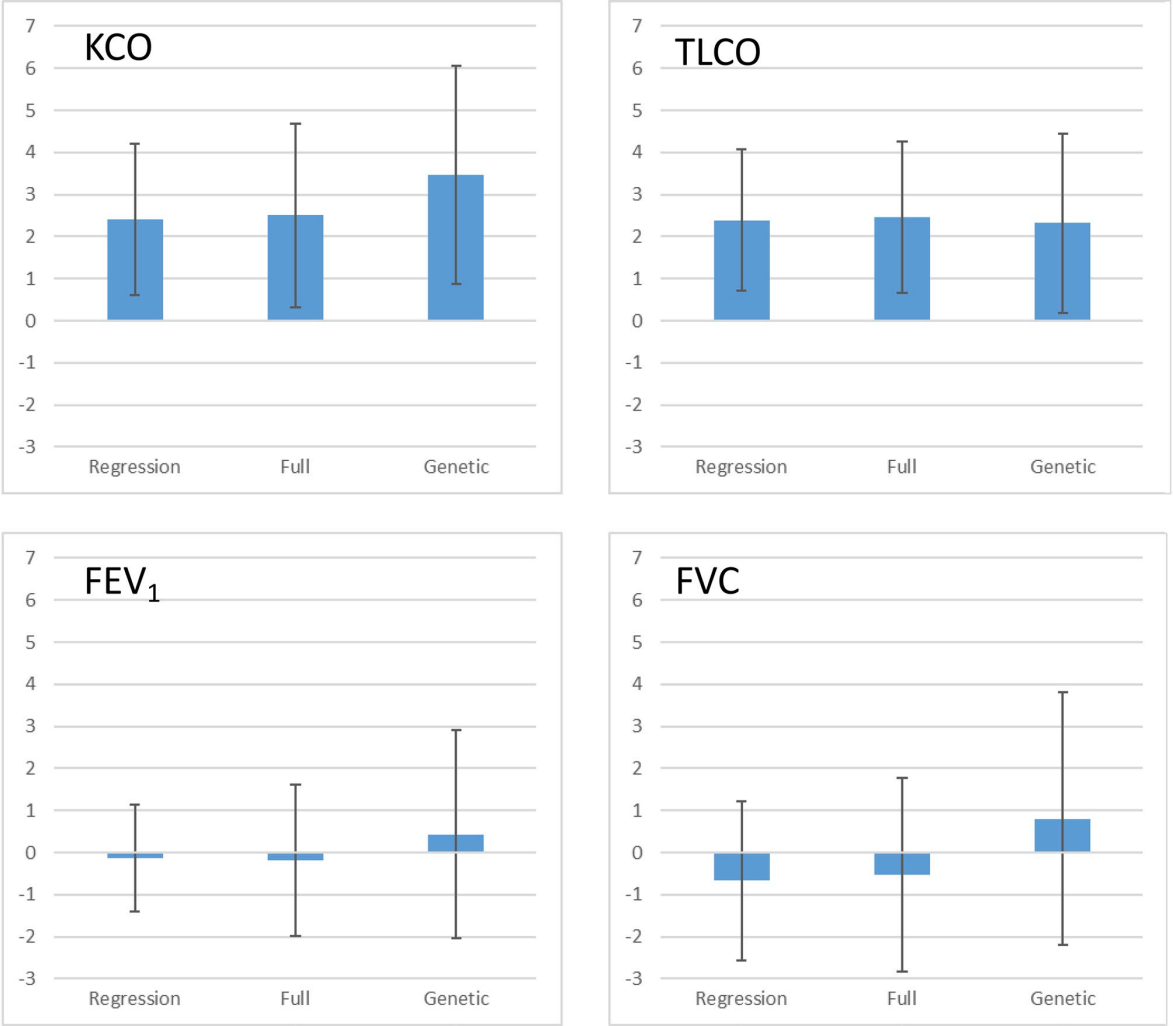
Association of metformin with annual changes in lung function (mean and 95%-confidence intervals). The four panels refer to KCO, TLCO, FEV_1_ and FVC, and the changes on the vertical axes are expressed as percent predicted at baseline. Positive values mean that the fall of the lung function measure (negative change) is reduced by the respective amount compared to the non-metformin group. Each panel shows three estimated effects, first from the regression analyses, then from the propensity score analyses using either full or genetic matching. As can be seen, the results were similar within each lung function measure and the pattern of statistically significant vs non-significant effects was the same. In addition, indicators for collinearity are given. Results for any metformin therapy as well as for TLCO were similar (see “Results” section and Tables S1, S2, S3).

The above-mentioned analyses were repeated using propensity scores for full and genetic matching. There were no significant effects of metformin on the annual decline of FEV_1_ with both full and genetic matching (*p* = 0.833 and *p* = 0.741, respectively). The same was true for FVC (*p* = 0.652 and *p* = 0.578). In contrast, the annual decline of KCO was associated with metformin therapy in both full (*p* = 0.0253) and genetic matching (*p* = 0.0470). For all covariates, the standardized mean difference between groups after matching was below 0.1, indicating excellent matching for both procedures. Similarly, the annual decline of TLCO was associated with metformin therapy in full and genetic matching (*p* = 0.0076 and *p* = 0.0315, respectively. The effect sizes of metformin therapy on the annual decline of FEV_1_, FVC, KCO and TLCO expressed as %predicted at baseline are shown in Fig. 2 and compared with those of the regression analyses.

### Sensitivity analyses

The linear regression analyses were repeated with HbA1c (n = 1498)as well as Hb (n = 1516) as additional predictors to account for potential effects of diabetes control. Their inclusion did not change the pattern of statistical significance. Moreover, the number of the final visit of each patient was included as additional categorical predictor (n = 1541). Again, the associations of metformin with KCO and TLCO remained statistically significant, while those with FEV_1_ and FVC were still not significant. The additional introduction of respiratory therapy (any LABA, any LAMA, any ICS) did also not affect the significant results for KCO and TLCO (n = 1541). The same was true when introducing cardiac medication (ACE/ARB inhibitors) as additional covariate (n = 1541).

## Discussion

In the present study we observed significant associations between metformin treatment for diabetes and the annual decline of lung diffusing capacity in patients with COPD. This was true for KCO and TLCO, whereas no associations with spirometric lung function (FEV_1_ and FVC) were found. To account for differences in baseline conditions, a number of baseline parameters such as FEV_1_ and pack-years were included as covariates. The observations suggest an association of metformin intake with lesser decline in the capability of gas uptake in terms of diffusing capacity, a measure commonly taken as functional marker of lung emphysema^30^. While emphysema is considered to be linked to premature aging^31^, metformin is discussed as potential anti-aging medication^18,32^. Combining these results, our data suggest an association of metformin with an anti-aging effect on the lung of patients with stable COPD. This finding is in line with recently published data using CT imaging and reporting a beneficial effect of metformin regarding lung emphysema^19^.

There are several links between COPD and aging. With increasing life expectancy the prevalence of COPD raises even if risk factors remain constant^33^. Moreover, there is evidence linking chronic diseases, especially COPD, to premature aging^1,2^. This has been demonstrated in terms of cellular senescence for different cell types^6,34,35^, biomarkers of the lung^36^, telomere length of blood leucocytes, and even phenomenological markers such as skin texture^37,38^. Animal models have lend support to this view, using cigarette smoke exposure to elicit lung emphysema^39,40^, as well as demonstrating that changes in the pulmonary capillary bed are essential in the development of emphysema^41^. These mechanisms cannot be experimentally addressed in patients but suggest a reverse approach by investigating potential anti-aging effects of drugs on the lung using surrogate markers of lung emphysema, among which CO diffusing capacity has the advantage of being easily monitored over time without repeated radiation exposures. We used this measure, both as total diffusing capacity (TLCO) and value per unit lung volume (KCO), to quantify the associations with metformin.

The exploration of therapeutic anti-aging approaches has gained much interest over time, and a panel of candidate drugs and interventions has been proposed or investigated^3,36^. Most of these are of experimental nature or taken by few patients, with the consequence that data in humans are rare or not available. Metformin has the advantage of being widely used for the treatment of diabetes^32^, a frequent comorbidity in COPD^12^. Epidemiological, preclinical and clinical studies have shown that metformin, in addition to achieving glycaemic control, has positive effects against tumour development and recurrence^42^, as well as protective effects in cardiovascular^43^, neurodegenerative^44^ and autoimmune diseases^45^. Metformin also has beneficial properties, including anti-inflammatory effects, positive effects on microvasculature, membrane homeostasis and anti-apoptotic effects^46^. Recent data also show positive effects on the overall aging process^32^.

The main metabolic effects of metformin are triggered via activation of AMPK and inhibition of complex I of mitochondrial electron transport chain^47^. There are also direct effects on mTORC1, PGC1-alpha, Insulin-IGF1 signalling, SIRT1, NF-kappaB signalling, and pro-inflammatory cytokines^48^. Thus, metformin can modulate multiple metabolic and cellular processes associated with the development of age-related diseases, including inflammation, autophagy and cellular senescence^48^. The positive effects of metformin are reflected in experimental observations that it can increase the average lifespan of nematodes by about 57 percent, as well as that of mice and rats^48^. It is still an open question whether metformin can also delay “old-age diseases” in humans without diabetes. This question is addressed in the TAME (Targeting Aging with Metformin) study, planned at the Institute for Aging Research at the Albert Einstein College of Medicine, New York City^49^. This phase III study aims to assess whether metformin can prolong life and support healthy aging, which might also be relevant for the development and course of COPD. Importantly, a recent longitudinal analysis of data from COPDgene using CT imaging directly showed a reduction in the progression of emphysema in patients taking metformin^19^.

In line with this, our observation of a reduced annual decline of lung diffusing capacity suggests an association of metformin with anti-aging effects on the progression of emphysema. As an alternative, the association could be attributed to a common genotype underlying a COPD phenotype with less emphysema and the development of diabetes. This hypothesis has already been discussed in a radiological study describing diabetes as a risk factor for obstructive airway disease but not emphysema^13^. We additionally proposed the possibility of beneficial effects of anti-diabetes medication in a cross-sectional analysis of patients with diabetes and COPD, with focus on diffusing capacity^12^. The present findings on the changes of diffusing capacity over time seem to support the hypothesis of a genuine association with diabetes medication, specifically metformin.

The lack of association of metformin with changes of FEV_1_, in contrast to diffusing capacity, might be explained by the assumption that anti-aging effects of metformin occur at the level of capillaries and thus are manifest in diffusing capacity, in accordance with the known beneficial effects of metformin on microvasculature^46^ and the link between emphysema and damage of the pulmonary capillary bed^41^. In contrast, FEV_1_ reflects overall mechanical alterations comprising changes in both lung parenchyma and conducting airways and might thus be less suitable for the assessment of anti-aging effects. In line with this, impairments of spirometry that are often interpreted as reflecting “lung age” seem to be independent from alterations of lung diffusing capacity in the elderly^50^. Cardiovascular diseases which are known to be linked to impaired diffusing capacity were even more frequent in the metformin group, and pack-years were greater, while no association with respiratory or cardiac therapy was detectable; thus, the only remaining explanation in terms of medication appeared to be metformin.

### Limitations and strengths

The evidence for the observed association of metformin with diffusing capacity in COPD patients was indirect and based on a retrospective observational analysis, not an interventional study. We used diffusing capacity as a proxy for emphysema, as CT images were available only in a minority of patients. Another limitation of the study is the low number of patients with diabetes mellitus and continuous use of metformin across all follow-up visits (n = 76), compared with a relatively high number of non-diabetic patients (n = 1465). The low proportion of diabetic patients is based on the strict definition of this group. Diabetes patients with diet-only treatment recommendations, as well as diabetics with other anti-diabetic therapies, were excluded to identify the association between lung function and metformin as clearly as possible. On the other hand, this favoured the use of propensity score analysis, as there were many patients in the control available that could be matched to the metformin group. To identify the effect as reliably as possible, we included many covariates and used three statistical procedures in parallel; their results were fully consistent with each other. As shown in the sensitivity analyses including the number of the last visit, our findings did not appear to depend on the loss of patients over time. The patients excluded could not serve as a proper control group as many of them had metformin at one visit but not all visits, and the number of patients without any metformin was too small for the purpose of a control group. We thus used the large and diverse group of patients without diabetes as a reference group and aimed to account for the severity of COPD and for comorbidities by several matching procedures. Although adherence to the intake of metformin was not assessed, we relied on previous results demonstrating a very high degree of adherence to both inhaled and oral medication in COSYCONET patients^51^. Of course, the results should be checked in other large observational COPD cohorts in which systemic medication is recorded, in addition to randomised trials^49^.

## Conclusion

Using longitudinal data from a large COPD cohort, we found that treatment with the anti-diabetic drug metformin was associated with a reduced annual decline of CO diffusing capacity. As diffusing capacity is a marker of lung emphysema and emphysema thought to be linked to premature aging, our observations are in line with anti-aging and anti-emphysema effects of metformin that have been observed in cell-culture, animal experiments and in human subjects using CT imaging. Our study provides further evidence for a protective association of metformin with lung emphysema based on an analysis of functional markers.

## Supplementary Information

Supplementary Figure S1.

Supplementary Tables.

## Data Availability

The basic data are part of the German COPD cohort COSYCONET (www.asconet.net/) and available upon request. There is a detailed procedure for this on the website of this network. Specifically, the data can be obtained by submission of a proposal that is evaluated by the steering committee. All results to which the manuscript refers, are documented appropriately in the text, figures or tables.

## Abbreviations

AMPK: Adenosine monophosphate-activated protein kinase
COPD: Chronic obstructive pulmonary disease
CO: Carbon monoxide
CRP: C-reactive protein
FEV_1_: Forced expiratory volume in 1 s
FVC: Forced vital capacity
HbA1c: Glycated haemoglobin
Hb: Haemoglobin
KCO: Transfer coefficient
PI3K: Phosphoinositide 3-kinase
mMRC: Modified Medical Research Council Dyspnoea Score
mTOR: Mammalian Target of Rapamycin
SASP: Senescence-associated secretory profiles
TLCO: Diffusing capacity for carbon monoxide
