# Supplementary figures and images for: Reduced decline of lung diffusing capacity in COPD patients with diabetes and metformin treatment
Source: Sci Rep. 2022 Jan 26;12:1435. doi: 10.1038/s41598-022-05276-x (PMC8792053; doi:10.1038/s41598-022-05276-x)

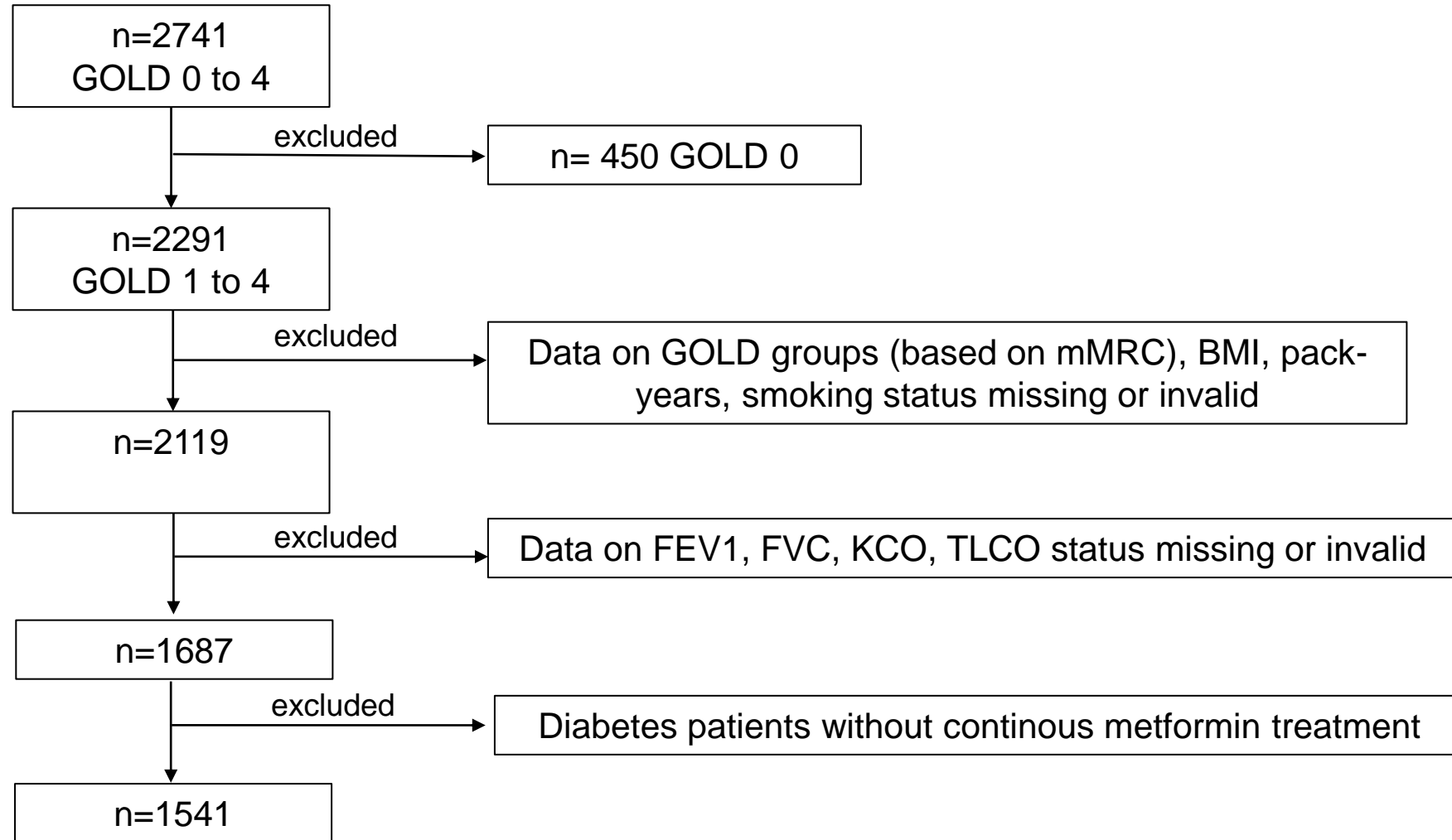

**Figure S1:** Flow chart of the selection of patients for the final analysis

Supplement: Supplementary file 1 — Supplementary Figure S1. [file 41598_2022_5276_MOESM1_ESM.pdf]
