# Supplementary material for: Reduced decline of lung diffusing capacity in COPD patients with diabetes and metformin treatment
Source: Sci Rep. 2022 Jan 26;12:1435. doi: 10.1038/s41598-022-05276-x (PMC8792053; doi:10.1038/s41598-022-05276-x)
Supplement: Supplementary file 2 — Supplementary Tables. [file 41598_2022_5276_MOESM2_ESM.docx]

# Supplement to:

**Reduced decline of lung diffusing capacity in COPD and diabetes with metformin treatment: Results from COSYCONET**

Kathrin Kahnert^1^, Stefan Andreas^2^, Christina Kellerer^3,4^, Johanna I. Lutter^5^, Tanja Lucke ^3^,
Önder Yildirim^6^, Mareike Lehmann^6^, Jochen Seissler^7^, Jürgen Behr^1^, Marion Frankenberger^6^, Robert Bals^8^, Henrik Watz^9^, Tobias Welte^10^, Franziska C. Trudzinski^11^, Claus F. Vogelmeier^12^, Peter Alter^12^, Rudolf A. Jörres^3^

# Legends to supplemental figure

**Figure S1:**

Flow chart of the selection of patients for the final analysis

**Supplemental Tables**

|  | | Non-standardized | | Standardized coefficient | p-value | 95%-Confidence interval for B | | Collinearity  VIF |
| --- | --- | --- | --- | --- | --- | --- | --- | --- |
|  |  | Regression coefficient B | SE | Beta |  | Lower | Upper |  |
|  | Sex (female vs. male) | 0.078 | 0.393 | 0.005 | 0.842 | -0.692 | 0.849 | 1.126 |
|  | Age (y) | -0.085 | 0.023 | -0.094 | 0.000 | -0.130 | -0.039 | 1.111 |
|  | BMI (kg/m²) | 0.183 | 0.040 | 0.123 | <0.001 | 0.106 | 0.261 | 1.213 |
|  | Pack-years | -0.021 | 0.005 | -0.100 | <0.001 | -0.031 | -0.010 | 1.157 |
|  | Smoking status (active) | -0.871 | 0.441 | -0.052 | 0.049 | -1.737 | -0.005 | 1.169 |
|  | Symptoms (GOLD BD vs AC) | -1.372 | 0.413 | -0.091 | 0.001 | -2.183 | -0.561 | 1.289 |
|  | Exacerbations (GOLD CD vs AB) | 0.410 | 0.402 | 0.026 | 0.307 | -0.378 | 1.199 | 1.094 |
|  | Cardiovascular disease* | 0.142 | 0.479 | 0.007 | 0.767 | -0.797 | 1.081 | 1.072 |
|  | FEV_1_ %predicted baseline | 0.117 | 0.013 | 0.282 | <0.001 | 0.092 | 0.142 | 1.641 |
|  | TLCO %predicted baseline | -0.130 | 0.011 | -0.364 | <0.001 | -0.152 | -0.108 | 1.659 |
|  | Metformin therapy (continuous) | 2.392 | 0.856 | 0.069 | 0.005 | 0.713 | 4.072 | 1.053 |

**Table S1. Association between annual decline of TLCO %predicted and metformin monotherapy.** The table shows the results of multivariate linear regression analysis in terms of the non-standardized regression coefficients, their standard errors (SE), and 95%-confidence intervals, and the standardized coefficients. All clinical and functional indices refer to baseline (visit 1), the change of TLCO to that between baseline and each patient’s last visit, expressed as %predicted at baseline. Additionally, the Variance Inflation Factor (VIF) from the collinearity diagnostics in SPSS is given, indicating that there was no problem with collinearity as all values were close to 1. *The diagnosis of cardiovascular disease comprised heart failure, coronary artery disease and myocardial infarction. In addition, indicators for collinearity are given. Results for any metformin therapy as well as for KCO were similar (see Results and Table 2)

|  | | Non-standardized | | Standardized coefficient | p-value | 95%-Confidence interval for B | | Collinearity  VIF |
| --- | --- | --- | --- | --- | --- | --- | --- | --- |
|  |  | Regression coefficient B | SE | Beta |  | Lower | Upper |  |
|  | Sex (female vs. male) | 0.541 | 0.296 | 0.049 | 0.068 | -0.039 | 1.120 | 1.116 |
|  | Age (y) | 0.000 | 0.017 | -0.001 | 0.980 | -0.034 | 0.034 | 1.104 |
|  | BMI (kg/m²) | 0.086 | 0.029 | 0.080 | 0.003 | 0.030 | 0.142 | 1.110 |
|  | Pack-years | -0.004 | 0.004 | -0.026 | 0.341 | -0.012 | 0.004 | 1.137 |
|  | Smoking status (active) | -0.803 | 0.332 | -0.066 | 0.016 | -1.455 | -0.151 | 1.161 |
|  | Symptoms (GOLD BD vs AC) | -0.334 | 0.308 | -0.031 | 0.278 | -0.937 | 0.270 | 1.251 |
|  | Exacerbations (GOLD CD vs AB) | 0.055 | 0.304 | 0.005 | 0.855 | -0.540 | 0.651 | 1.093 |
|  | Cardiovascular disease* | 0.185 | 0.361 | 0.013 | 0.608 | -0.523 | 0.894 | 1.071 |
|  | FEV_1_ %predicted baseline | -0.028 | 0.009 | -0.094 | 0.001 | -0.045 | -0.011 | 1.262 |
|  | Metformin therapy (continuous) | -0.145 | 0.647 | -0.006 | 0.823 | -1.414 | 1.124 | 1.053 |

**Table S2. Association between annual decline of FEV_1_ %predicted and metformin monotherapy.** The table shows the results of multivariate linear regression analysis in terms of the non-standardized regression coefficients, their standard errors (SE), and 95%-confidence intervals, and the standardized coefficients. All clinical and functional indices refer to baseline (visit 1), the change of FEV_1_ to that between baseline and each patient’s last visit, expressed as %predicted at baseline. Additionally, the Variance Inflation Factor (VIF) from the collinearity diagnostics in SPSS is given, indicating that there was no problem with collinearity as all values were close to 1. *The diagnosis of cardiovascular disease comprised heart failure, coronary artery disease and myocardial infarction. In addition, indicators for collinearity are given. Results for any metformin therapy as well as for FVC were similar (see Results and Table S3)

|  | | Non-standardized | | Standardized coefficient | p-value | 95%-Confidence interval for B | | Collinearity  VIF |
| --- | --- | --- | --- | --- | --- | --- | --- | --- |
|  |  | Regression coefficient B | SE | Beta |  | Lower | Upper |  |
|  | Sex (female vs. male) | 0.098 | 0.443 | 0.006 | 0.825 | -0.771 | 0.967 | 1.116 |
|  | Age (y) | 0.000 | 0.026 | 0.000 | 0.996 | -0.051 | 0.051 | 1.104 |
|  | BMI (kg/m²) | 0.057 | 0.043 | 0.036 | 0.182 | -0.027 | 0.141 | 1.110 |
|  | Pack-years | -0.005 | 0.006 | -0.022 | 0.427 | -0.017 | 0.007 | 1.137 |
|  | Smoking status (active) | 0.656 | 0.498 | 0.036 | 0.188 | -0.321 | 1.632 | 1.161 |
|  | Symptoms (GOLD BD vs AC) | -0.880 | 0.461 | -0.054 | 0.056 | -1.784 | 0.024 | 1.251 |
|  | Exacerbations (GOLD CD vs AB) | 0.027 | 0.455 | 0.002 | 0.953 | -0.865 | 0.919 | 1.093 |
|  | Cardiovascular disease* | 0.241 | 0.541 | 0.012 | 0.657 | -0.822 | 1.303 | 1.071 |
|  | FEV_1_ %predicted baseline | -0.007 | 0.013 | -0.017 | 0.559 | -0.032 | 0.018 | 1.262 |
|  | Metformin therapy (continuous) | -0.673 | 0.969 | -0.018 | 0.487 | -2.575 | 1.228 | 1.053 |

**Table S3. Association between annual decline of FVC_1_ %predicted and metformin monotherapy.** The table shows the results of multivariate linear regression analysis in terms of the non-standardized regression coefficients, their standard errors (SE), and 95%-confidence intervals, and the standardized coefficients. All clinical and functional indices refer to baseline (visit 1), the change of FVC_1_ to that between baseline and each patient’s last visit, expressed as %predicted at baseline. Baseline FEV_1_ was chosen as predictor instead of baseline FVC in order to ensure comparability to Table S2. Results for baseline FVC were virtually identical. Additionally, the Variance Inflation Factor (VIF) from the collinearity diagnostics in SPSS is given, indicating that there was no problem with collinearity as all values were close to 1. *The diagnosis of cardiovascular disease comprised heart failure, coronary artery disease and myocardial infarction. In addition, indicators for collinearity are given. Results for any metformin therapy as well as for FEV_1_ were similar (see Results and Table S2)
